# Sero-epidemiological and functional evaluation of IgG specific for a cysteine protease inhibitor of *Plasmodium falciparum*, falstatin

**DOI:** 10.1128/iai.00202-26

**Published:** 2026-08-04

**Authors:** Jyoti Bhardwaj, Erik L. Gaskin, Prasida Holla, Oscar De Los Santos, Safiatou Doumbo, Kassoum Kayentao, Aissata Ongoiba, Boubacar Traore, Peter D. Crompton, Tuan M. Tran

**Affiliations:** 1 Division of Infectious Diseases, Department of Medicine, Indiana University School of Medicine 12250, Indianapolis, Indiana, USA; 2 Division of Pediatric Infectious Diseases and Global Health, Department of Pediatrics, Indiana University School of Medicine, Indianapolis, Indiana, USA; 3 Mali International Center of Excellence in Research, University of Sciences, Technique and Technology of Bamako, Bamako, Mali; 4 Malaria Infection Biology and Immunity Section, Laboratory of Immunogenetics, National Institute of Allergy and Infectious Diseases, National Institutes of Health, Rockville, Maryland, USA; University of California Davis, Davis, California, USA

**Keywords:** *Plasmodium falciparum*, malaria risk, prospective cohort, longitudinal study, falstatin, inhibitor of cysteine proteases, IgG antibodies

## Abstract

Recently approved malaria pre-erythrocytic vaccines are important public health tools for the reduction of malaria morbidity and mortality. However, these vaccines have shown reduced protection in some of the most vulnerable age groups, require multiple dosing, and have the potential for parasite escape. These limitations have motivated the development of vaccines targeting multiple stages of the parasite life cycle. To ensure a robust pipeline, pre-clinical evaluation of additional malaria vaccine candidate antigens remains a key priority. The *P. falciparum* cysteine protease inhibitor falstatin can limit the cysteine protease activity of falcipains, controlling proteolysis at the site of merozoite entry on erythrocytes, thereby facilitating parasite invasion. Antibodies against falstatin have been shown to reduce the ability of merozoites to invade erythrocytes, raising the possibility that naturally acquired antibody responses could confer a similar advantage to the host. Here, IgG responses to full-length falstatin (falstatin_FL_) and two smaller, conserved regions (falstatin_35–68_ and falstatin_289–335_) were determined at the pre-malaria season baseline in a prospective cohort study conducted in Kalifabougou, Mali. The presence of anti-falstatin IgG was not associated with reduced risk of clinical malaria overall. However, baseline falstatin seropositivity predicted a non-statistically significant reduction in risk of febrile malaria within the first 30 days after incident parasitemia (log-rank statistic 3.2, *P* = 0.072). Using *in vitro* growth inhibitory assays, neither affinity-purified anti-falstatin_289–335_ IgG nor affinity-purified anti-falstatin_FL_ IgG consistently inhibited *P. falciparum* blood-stage parasites. Larger cohort studies would be needed to confirm whether pre-existing anti-falstatin antibodies can delay the progression of malaria symptoms after blood-stage infection.

## INTRODUCTION

Malaria remains a global health burden with more than 280 million cases per year (1). Among the parasites responsible for human malaria, *Plasmodium falciparum* is the most lethal and is responsible for the vast majority of the ~610,000 malaria-related deaths (1). Young children living in sub-Saharan Africa are particularly vulnerable to the severe and life-threatening manifestations of malaria due to the lack of acquired immunity. After significant reductions in malaria cases owing to effective preventative measures, including insecticide-treated nets and malaria chemoprophylaxis, combined with prompt diagnosis and treatment, progress has stalled in recent years (1). The recently approved malaria pre-erythrocytic vaccines RTS,S/AS01 and R21/Matrix-M are important steps toward reducing malaria morbidity. However, protection afforded by these vaccines may be less protective in some of the most vulnerable age groups (e.g., infants <5 months of age, women of child-bearing potential) and require multiple dosing to maintain efficacy (2, 3). Furthermore, the current pre-erythrocytic vaccines are considered “leaky” in that they may not completely block blood-stage infection in settings of intense exposure to infectious mosquito bites (4). The potential for parasites escaping the host response to pre-erythrocytic vaccines to initiate blood-stage infection, thereby contributing to clinical disease and onward transmission, has motivated the development of vaccines targeting multiple stages of the parasite life cycle (5, 6). Indeed, vaccines targeting the blood-stage antigen *P. falciparum* reticulocyte-binding protein homolog 5 (PfRH5) have already advanced to phase 2 trials (7, 8). However, to ensure a robust pipeline, pre-clinical evaluation of additional malaria vaccine candidate antigens remains a key priority.

Current malaria vaccines target essential parasite ligands that bind host receptors to disrupt the critical steps of invasion of either hepatocytes or erythrocytes. Parasite inhibitors of cysteine proteases (ICPs), on the other hand, can indirectly facilitate parasite invasion by restricting proteolytic cleavage of essential invasion proteins at the site of host cell attachment. The *P. falciparum* protein falstatin was first characterized as an endogenous ICP that is initially expressed during the late intraerythrocytic stage (schizonts) and through the merozoite and early ring stages (9). After schizont rupture, falstatin is believed to limit the cysteine protease activity of falcipains to control proteolysis at the site of merozoite entry on a new erythrocyte, thereby abetting parasite invasion. Antibodies against falstatin have been shown to reduce the ability of merozoites to invade erythrocytes, possibly by allowing unchecked proteolysis of critical adhesion proteins (9). This suggests that targeting falstatin to inhibit its activity may act synergistically with vaccines that aim to disrupt parasite ligand-host receptor interactions. Here, we determine whether naturally acquired IgG antibody responses to falstatin are associated with protection against clinical malaria in a longitudinal cohort study of Malian children and adults. We also evaluate whether rabbit IgG antibodies raised against recombinant falstatin can inhibit parasite growth *in vitro*.

## RESULTS

### Study participants and study site

Participants in the current study included 578 children and adults from the 695 participants who were initially enrolled in the Kalifabougou prospective cohort study during its first year. This longitudinal study of malaria immunity has been ongoing since May 2011 in Kalifabougou, Mali, where malaria transmission is seasonal and intense (10). The participants included in the current study were limited to those who had plasma samples available for analysis (Table 1; Fig. S1A and B). The distributions of age, sex, and Hb genotypes, including sickle cell trait (HbAS), in the current study were representative of the parent cohort. However, participants who were PCR-negative for *P. falciparum* infection at enrollment (healthy baseline), particularly those with PfRH5-specific IgG responses, were underrepresented in the current study due to the exhaustive use of their samples during our prior studies that evaluated malaria risk in individuals (11–13) (Table 1; Fig. S1B).

**TABLE 1:** Study participant characteristics^*d*^

Characteristic	Current study *N* = 578	Full cohort *N* = 695	*P* value
Age (years)	7.0 (4.2) [0.2, 24.0]	7.5 (4.5) [0.2, 25.0]	0.081^*a*^
Sex			0.5^*b*^
Female	275 (48%)	344 (49%)	
Male	303 (52%)	351 (51%)	
*P. falciparum* PCR status at enrollment			0.011^*b*^
Negative	272 (47%)	377 (54%)	
Positive	306 (53%)	318 (46%)	
Hb genotype			>0.9^*c*^
AA	456 (79%)	552 (79%)	
AC	60 (10%)	70 (10%)	
AS	58 (10%)	69 (9.9%)	
CC	1 (0.2%)	1 (0.1%)	
SC	3 (0.5%)	3 (0.4%)	

^*a*^ Wilcoxon rank-sum test.

^*b*^ Pearson’s Chi-squared test.

^*c*^ Fisher’s exact test.

^*d*^ Mean (SD) [Min, Max]; *n* (%).

### Falstatin antigens

**Fig 1:**
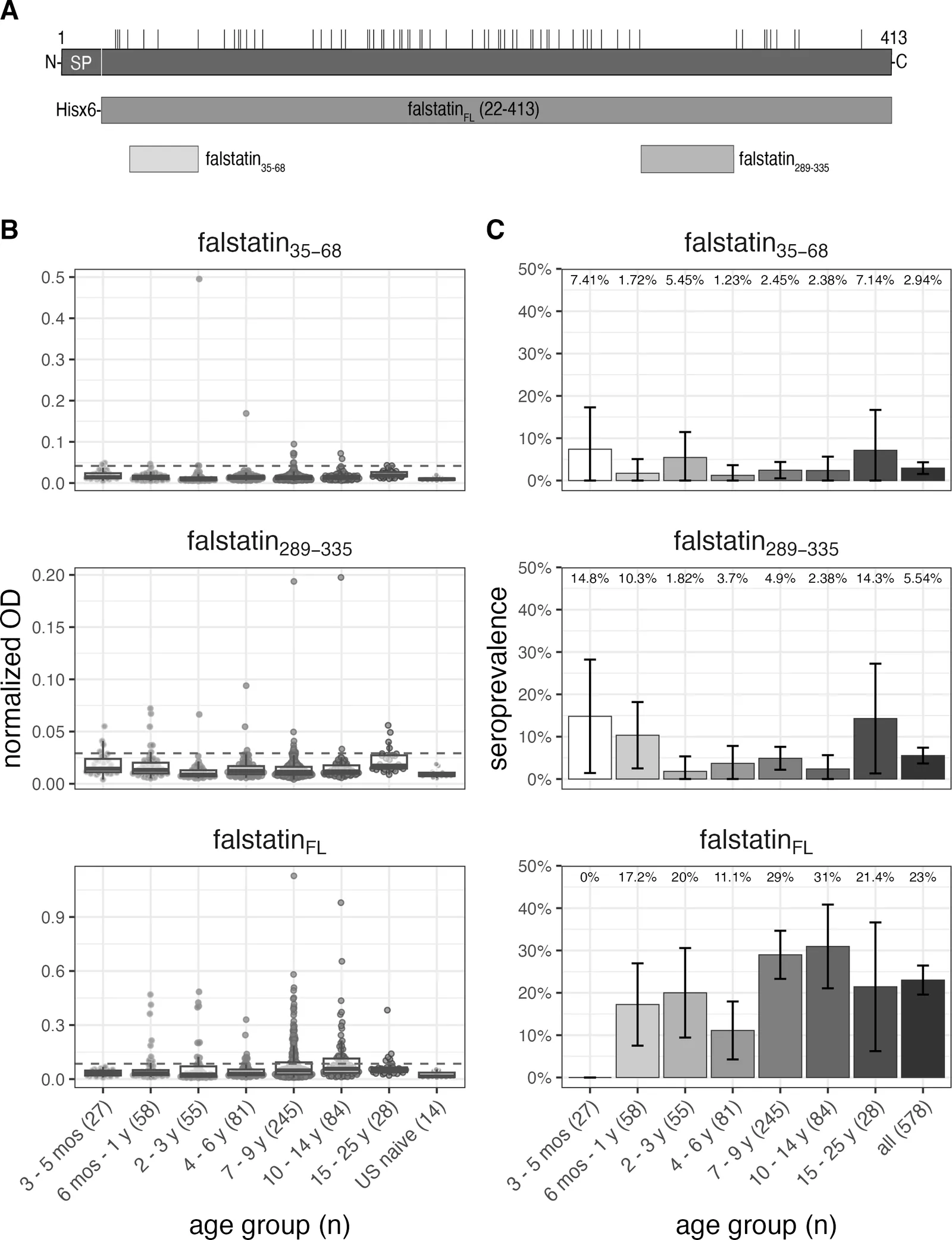
Plasma IgG reactivity to falstatin constructs in Malian children and adults. (**A**) Schematic of the *P. falciparum* falstatin protein with non-synonymous single-nucleotide polymorphisms indicated as tick marks. Below the schematic are regions expressed as recombinant, full-length protein (falstatin_FL_) in *Escherichia coli* or synthesized as smaller polypeptides (falstatin_35–68_ and falstatin_289–335_). SP, signal peptide. Hisx6, 6-histidine tag. (**B**) Plasma IgG reactivity against the indicated region by age group (y, years), measured by ELISA and shown as normalized optical density (OD). Box plots show the median and interquartile range (IQR) as the hinges, and whiskers indicate the smallest and largest values within 1.5 of the hinges. Dashed horizontal lines represent the thresholds for seropositivity, which is three standard deviations above the mean reactivity of 14 malaria-naive North American donors for each antigen. (**C**) Seroprevalence for each falstatin region by age group and among all individuals in the Kalifabougou cohort with available plasma. A seropositive response was defined as having an OD greater than the antigen-specific thresholds shown in panel **B**. Error bars are 95% confidence intervals using a normal distribution approximation.

To assess falstatin-specific serological and functional responses, falstatin was expressed as a full-length recombinant protein (falstatin_FL_) and synthesized as polypeptides spanning N-terminal and C-terminal regions, which were determined to be conserved by analysis of *P. falciparum* field isolates from the MalariaGEN *P. falciparum* Community Project variant catalog (14) (falstatin_35–68_ and falstatin_289–335_, respectively; Fig. 1A; Fig. S2). Importantly, the conserved falstatin_289–335_ region overlaps significantly with the BC loop region (falstatin residues 283–293), which has been shown to interact with cysteine proteases to inhibit their function (15).

### Falstatin-specific IgG reactivity and proportion of positive responders across age groups

Using plasma collected from the Kalifabougou cohort at enrollment in May 2011, pre-malaria season baseline IgG responses specific to falstatin_35–68_, falstatin_289–335_, and falstatin_FL_ were determined by ELISA. Overall IgG reactivities against falstatin_35–68_ and falstatin_289–335_ were low relative to falstatin_FL_, suggesting that these conserved linear epitopes may not be naturally immunogenic (Fig. 1B). There was a weak but significantly positive correlation between age and IgG reactivity against falstatin_FL_, with an initial monotonic increase in reactivity from 2 to 14 years of age, followed by a decrease in reactivity in the 15- to 25-year-old group (Fig. 1B; Fig. S3). By contrast, IgG reactivities against falstatin_35–68_ and falstatin_289–335_ did not correlate with age, being highest in the youngest and oldest age groups (Fig. 1B), with reactivity in the youngest age group likely reflecting maternal IgG. By seroprevalence, the overall percentage of positive responders was highest for falstatin_FL_ (23%), followed by falstatin_289–335_ (5.54%) and falstatin_35–68_ (2.94%; Fig. 1C). For falstatin_FL_, the highest point seroprevalence was among children aged 10–14 years (31%), whereas for falstatin_289–335_ and falstatin_35–68_, point seroprevalence was highest among infants <6 months (7.41% and 14.8%, respectively) and older participants aged 15–25 (7.14% and 14.3%, respectively; Fig. 1C), albeit with wide 95% confidence intervals. Among individuals who were IgG seropositive, subclass-specific responses skewed toward IgG3, particularly for falstatin_FL_, where 58% of the IgG positive responses were IgG3 (Fig. S4).

### Antibody responses to falstatin and malaria protection

**Fig 2:**
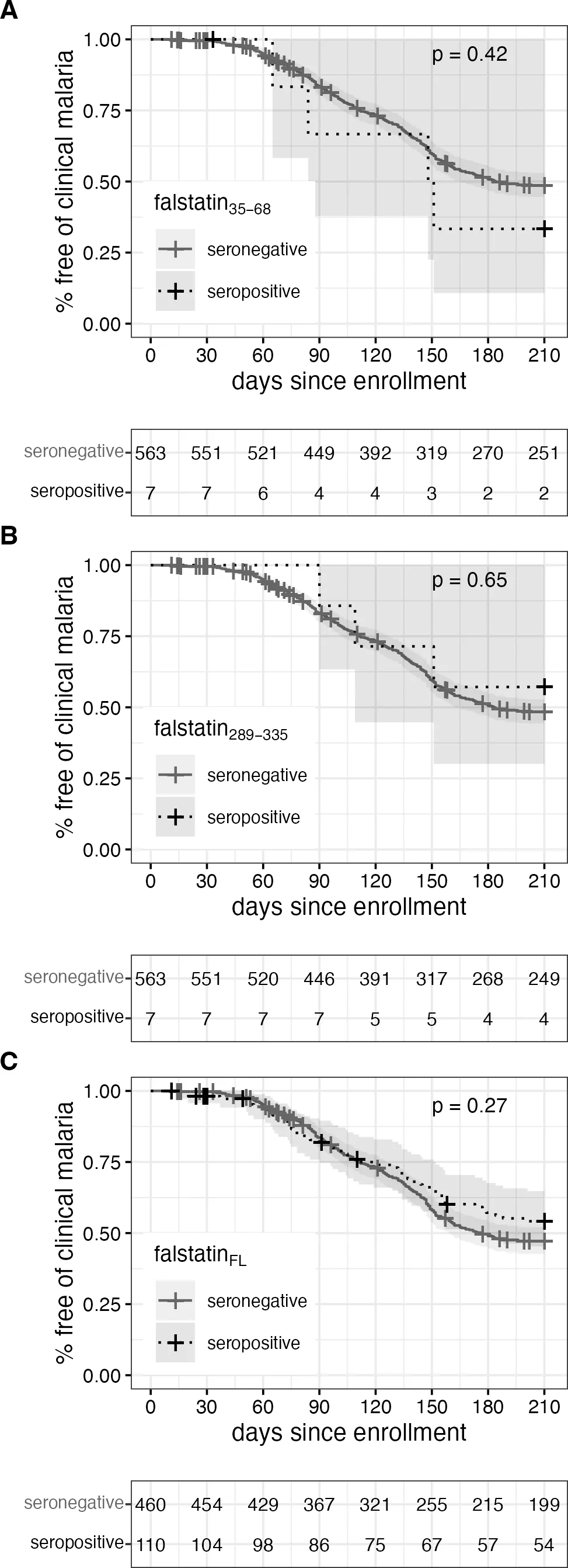
Time to first clinical malaria episode by baseline falstatin-specific IgG seropositivity. Kaplan–Meier plots by IgG seropositive status for (**A**) falstatin_35–68_, (**B**) falstatin_289–335_, and (**C**) falstatin_FL_. Significance was determined by log-rank analysis. Clinical malaria was defined as the presence of fever (axillary temperature ≥37.5°C) and a parasite density >2,500 parasites/µL by microscopy.

**Fig 3:**
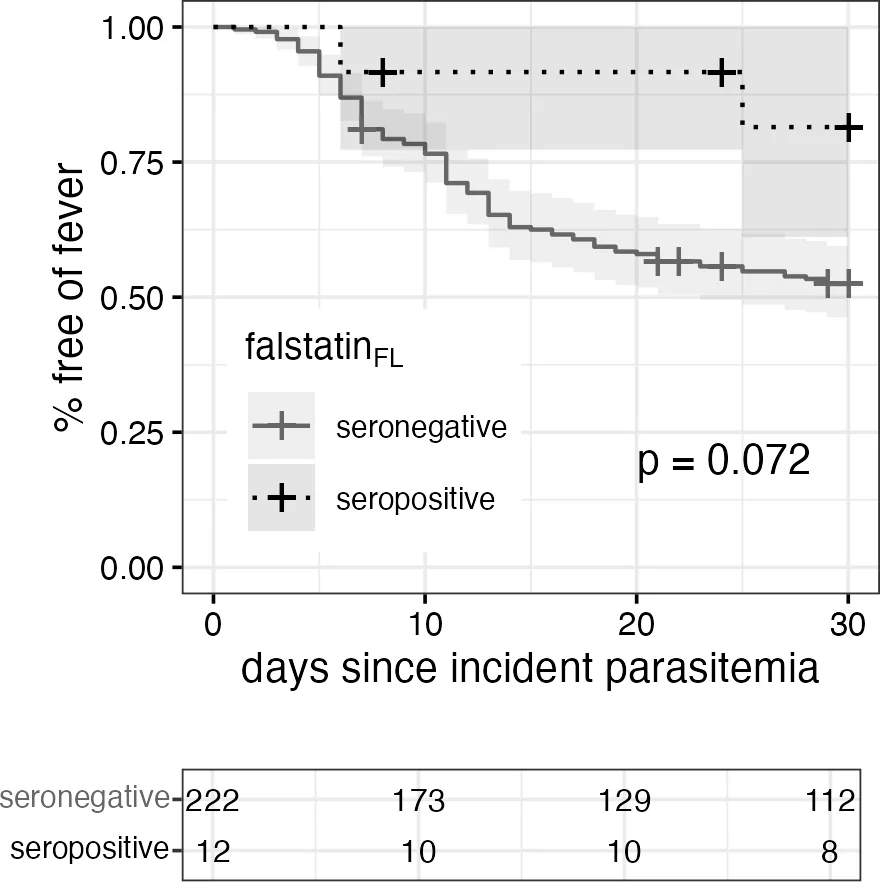
Time to first fever within the first 30 days after incident parasitemia by baseline falstatin-specific IgG seropositivity. Kaplan–Meier plots by IgG seropositive status for falstatin_FL_ for individuals who began the malaria season negative for *P. falciparum* by PCR. Fever was defined as the presence of axillary temperature ≥37.5°C after the first PCR- or microscopy-confirmed *P. falciparum* infection of the malaria season. Significance was determined by log-rank analysis.

To determine whether IgG responses to falstatin were associated with protection from clinical malaria, we used time-to-event analysis with baseline seropositivity to each of the falstatin antigens (determined in Fig. 1C) as predictors. There was no association between baseline seropositivity to any of the falstatin antigens and the prospective risk of clinical malaria by log-rank analysis (Fig. 2). Moreover, there was no association between falstatin_FL_ seropositivity and the risk of clinical malaria in a Cox proportional hazards model that included potential confounders, including age, *P. falciparum* PCR status at enrollment (a surrogate marker of prior malaria immunity), and the malaria-protective HbAS genotype as covariates (Table 2). To reduce possible confounding due to heterogeneous exposure to infectious mosquito bites (13, 16), we compared the time from incident parasitemia to febrile malaria between falstatin_FL_-seropositive and falstatin_FL_-seronegative individuals among those who were *P. falciparum* PCR-negative at the start of the malaria season. Analysis was restricted to 30 days from incident parasitemia to avoid capturing febrile episodes resulting from superinfection events (13). Baseline seropositivity to falstatin_FL_ predicted a reduced risk of progression to febrile malaria within the first 30 days after incident parasitemia by log-rank analysis (*χ*^2^ statistic = 3.2, *P* = 0.072). However, this result did not reach statistical significance (*P* < 0.05), most likely due to the small number of falstatin_FL_-seropositive individuals who were also *P. falciparum*-negative at enrollment (*n* = 12, Fig. 3). Nevertheless, the relationship between falstatin_FL_ seropositivity and reduced malaria risk was still maintained with inclusion of age, HbAS genotype, and IgG seropositivity to PfRH5 (hazard ratio, 0.48; 95% confidence interval [CI], 0.12–1.98; Table 3), the latter determined by a prior study (11).

**TABLE 2:** Baseline falstatin IgG seropositivity and risk of clinical malaria during the 2011 malaria season^*a*^^,^^*b*^

Covariate	*N*	Number of events	HR	95% CI	*P* value
Age (per year increase)	570	279	0.99	0.96, 1.02	0.4
*P. falciparum* PCR status at enrollment					
Negative	267	159	—	—	
Positive	303	120	0.58	0.43, 0.76	<0.001
Hb genotype					
Non-HbAS	512	256	—	—	
HbAS	58	23	0.68	0.44, 1.07	0.094
Falstatin_FL_					
Seronegative	460	232	—	—	
Seropositive	110	47	1.09	0.78, 1.53	0.6

^*a*^ Shown are hazard ratios (HR) and 95% confidence intervals (CI) for a Cox proportional hazards model with baseline falstatin IgG seropositivity, age, *P. falciparum* PCR status at enrollment, and sickle cell trait (HbAS) as covariates. For this analysis, follow-up time was 210 days from enrollment. Clinical malaria was defined as the presence of fever (axillary temperature ≥37.5°C) and parasite density >2,500 parasites/µL by microscopy.

^*b*^ CI, confidence interval; HR, hazard ratio.

**TABLE 3:** Baseline falstatin IgG seropositivity and risk of febrile malaria within the first 30 days after incident parasitemia^*a*^^,^^*b*^

Covariate	*N*	Number of events	HR	95% CI	*P* value
Age (per year increase)	224	106	0.97	0.92, 1.02	0.2
Hb genotype					
Non-HbAS	197	99	—	—	
HbAS	27	7	0.45	0.21, 0.97	0.043
Falstatin_FL_					
Seronegative	215	104	—	—	
Seropositive	9	2	0.48	0.12, 1.98	0.3
PfRH5					
Seronegative	213	104	—	—	
Seropositive	11	2	0.34	0.08, 1.39	0.13

^*a*^ Shown are hazard ratios (HR) and 95% confidence intervals (CI) for a Cox proportional hazards model with baseline falstatin IgG seropositivity, age, sickle cell trait (HbAS), and seropositivity to the blood-stage antigen PfRH5 as covariates. Analysis was limited to participants who began the 2011 malaria season negative for malaria infection by PCR. Start time was the time of incident parasitemia, defined as the midpoint between the last known PCR-negative event and first PCR-confirmed *P. falciparum* infection irrespective of symptoms. Clinical malaria was defined as the presence of fever (axillary temperature ≥37.5°C) and parasite density >2,500 parasites/µL by microscopy.

^*b*^ CI, confidence interval; HR, hazard ratio.

### Functional evaluation of falstatin-specific antibodies

**Fig 4:**
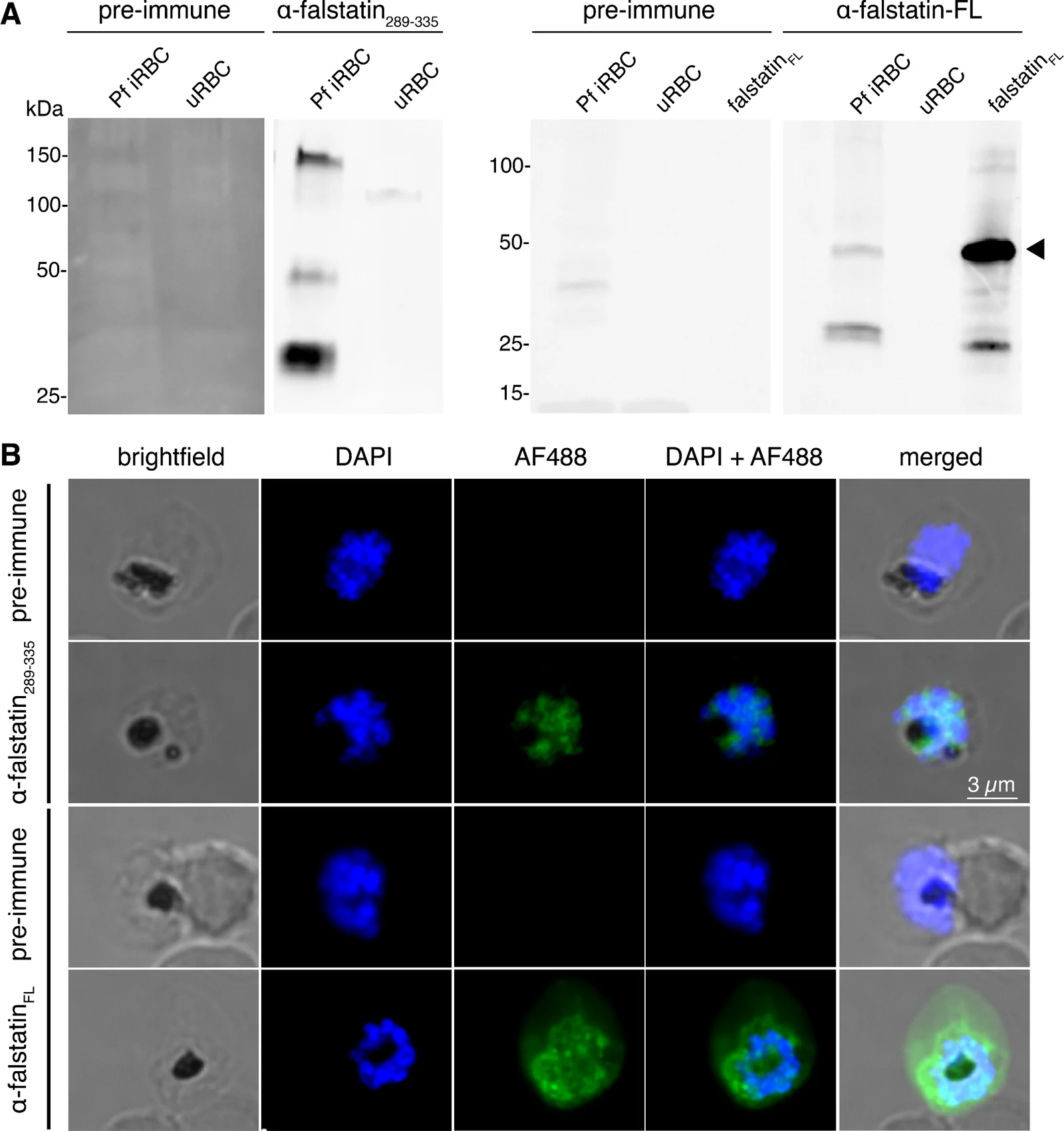
Detection of native *P. falciparum* falstatin by rabbit IgG raised against falstatin_289-335_ polypeptide and recombinant falstatin_FL_. (**A**) Immunoblots showing detection of falstatin in *P. falciparum*-infected red blood cell lysate (Pf iRBC), but not in uninfected RBC lysate (uRBCs), by IgG isolated from rabbits immunized with either falstatin_289–335_ or recombinant falstatin_FL_. Native falstatin and recombinant falstatin_FL_ migrate at the expected molecular weight of 47 kDa, as indicated by the arrow. Immunoblots using rabbit pre-immune sera are shown as a negative control for non-specific binding. MWM, molecular wt marker. (**B**) Immunofluorescence microscopy of *P. falciparum* blood-stage parasites using the same rabbit polyclonal IgG in A. *Plasmodium falciparum* (3D7) schizonts were stained with IgG purified from rabbit sera before (pre-immune) after immunization with the indicated falstatin antigen, followed by detection with an Alexa Fluor 488–conjugated anti-rabbit IgG secondary antibody (AF488, green). Nuclei were counterstained with DAPI (blue). Images were acquired using a 100× objective.

**Fig 5:**
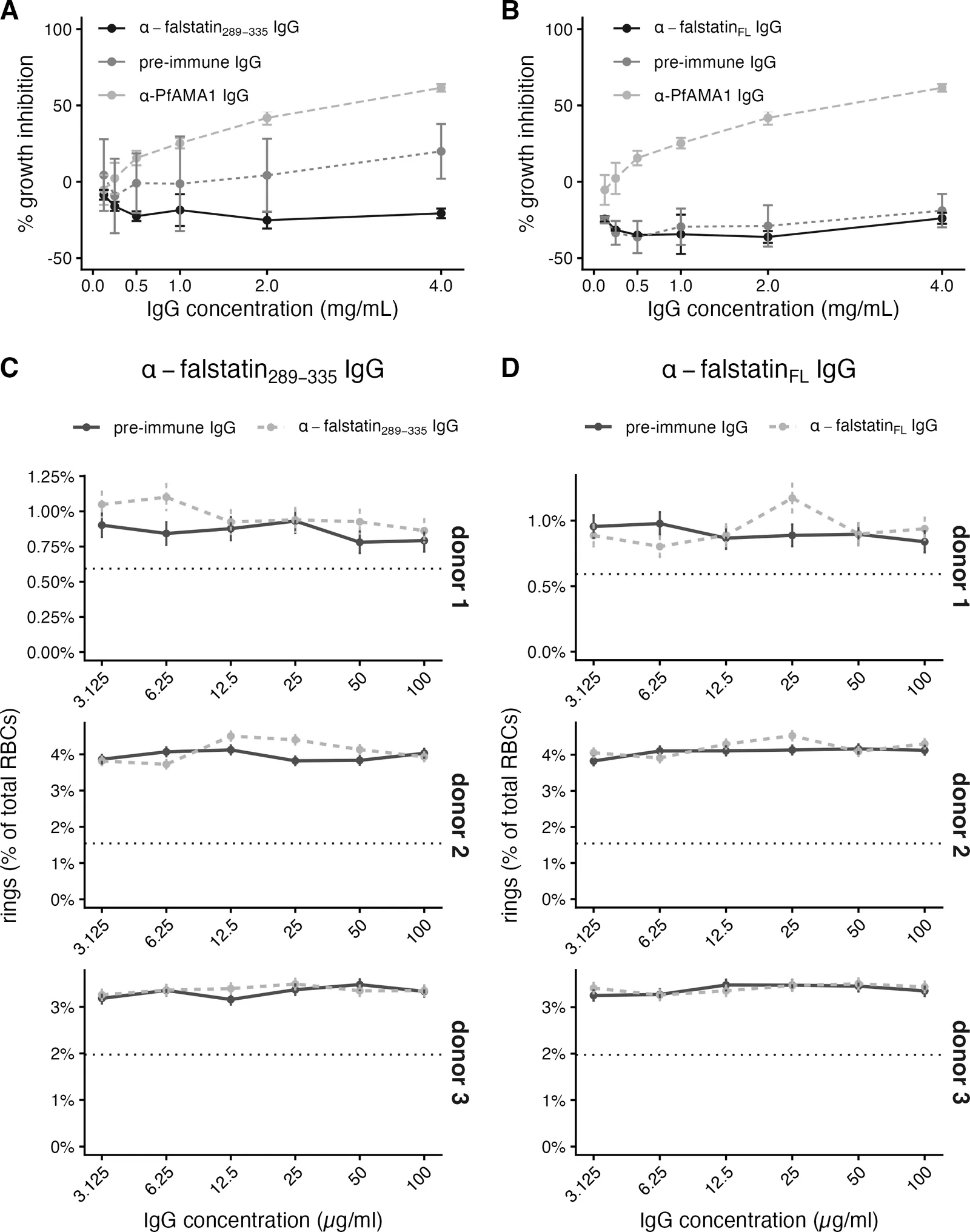
Functional evaluation of falstatin-specific IgG in *P. falciparum* growth and invasion assays. Parasite growth inhibition assays were performed using antigen-affinity purified IgG isolated from rabbits immunized with either (**A**) falstatin_289–335_ polypeptide or (**B**) recombinant falstatin_FL_. For each experiment, IgG purified from pre-immune sera was used as a non-specific negative control, and α-PfAMA1 IgG (total IgG purified from PfAMA-immunized rabbits) was used as a positive control. Error bars represent the standard error of the mean of triplicates. Data shown are representative of three independent experiments. Twenty-hour invasion inhibition assays were performed using IgG affinity purified against (**C**) falstatin_289–335_ polypeptide or (**D**) recombinant falstatin_FL_. Late trophozoite/early schizont-stage *P. falciparum* (3D7) cultures were incubated with serial two-fold dilutions of IgG for 20 h. Parasite invasion was assessed by quantifying ring-stage parasitemia using two-color flow cytometry (see Materials and Methods). Shown are the 95% confidence intervals using the normal approximation. Dotted horizontal line represents the percentage of rings for α-PfAMA1 IgG at 2 mg/mL. The data are from independent experiments, each using erythrocytes from a different donor.

Given the low natural immunogenicity of falstatin_35-68_ (<3%; Fig. 1C), functional evaluation of falstatin-specific antibodies was limited to falstatin_289–335_ and falstatin_FL_. Both affinity-purified anti-falstatin_289–335_ and anti-falstatin_FL_ polyclonal IgG isolated from the anti-sera of immunized rabbits reacted specifically with native falstatin by both immunoblot (Fig. 4A) and immunofluorescence microscopy (Fig. 4B). The detected protein migrated at the expected molecular weight of 47 kDa (albeit with additional lower molecular weight degradation products) and localized diffusely within schizonts (Fig. 4A and B), consistent with prior descriptions of falstatin (9, 15). Using standard *in vitro* growth inhibitory assays (GIA) (17), neither anti-falstatin_289–335_ nor anti-falstatin_FL_ polyclonal rabbit IgG inhibited *P. falciparum* (3D7) blood-stage parasites after ~40 h of culture at IgG concentrations up to 4 mg/mL (Fig. 5A and B). To better align with the methods from a prior study that showed anti-falstatin IgG inhibited erythrocyte invasion by *P. falciparum in vitro* (9), we performed 20-h invasion assays by flow cytometry and microscopy. Neither rabbit IgG specific for falstatin_289–335_ nor falstatin_FL_ at concentrations up to 100 µg/mL (twice the maximum concentration used by Pandey et al. [9]) reduced the proportion of ring-stage parasites when compared with pre-immune IgG controls by flow cytometry (Fig. 5C and D; Fig. S5). By microscopy, anti-falstatin IgG reduced new erythrocyte invasion relative to pre-immune IgG controls in only one out of three experiments (Fig. S6). Taken together, the data suggest that falstatin may not be a specific target of growth inhibitory IgG antibodies.

## DISCUSSION

Naturally acquired immunity to malaria develops over repeated infections, and parasite molecules critical for erythrocyte invasion are thought to be among the targets of acquired immunity (11, 18). Identifying such targets is essential for designing vaccines that can block blood-stage replication. Falstatin, a parasite inhibitor of cysteine proteases (ICPs), has been proposed as a potential target because it facilitates merozoite invasion by restricting proteolytic cleavage at the site of host cell entry (9). This prior study suggested that antibodies against falstatin could inhibit parasite invasion, raising the possibility that naturally acquired falstatin-specific antibodies might contribute to protection from clinical malaria (9). To investigate this, we evaluated antibody responses to both full-length and conserved regions of falstatin using functional assays and a longitudinal cohort study to determine whether these responses are associated with protective immunity.

Notably, the anti-falstatin_FL_ IgG seropositivity rate of 23% was lower than seropositivity rates for other *P. falciparum* blood-stage antigens that were previously evaluated in the Kalifabougou cohort (11, 12, 19). The exceptions are PfCyRPA and PfRH5. These two antigens (along with PfRipr, PfPTRAMP, and PfCSS) are members of the pentameric PfPCRCR complex that is essential for erythrocyte invasion (20–22) and demonstrated poor natural immunogenicity in this cohort, with respective seropositivity rates of <5% and 16% (11, 12). Despite this, anti-PfRH5 IgG still correlated with protection from febrile malaria and demonstrated growth inhibitory activity (11, 12), providing confidence that the Kalifabougou cohort can identify promising malaria vaccine candidates even when they are naturally less immunogenic (8). Naturally acquired IgG responses to falstatin were heavily skewed toward the IgG3 subclass, which implies a less durable antibody response given its shorter half-life relative to other subclasses (23). Thus, if advanced as a malaria vaccine candidate, a vaccine targeting falstatin would need to overcome its inherently poor natural immunogenicity and, ideally, also elicit responses favoring longer-lived IgG subclasses, such as IgG1.

The low falstatin seropositivity rates, which reduce the power to detect more moderate associations with the prospective risk of malaria, may explain the lack of statistically significant associations between falstatin-specific IgG responses and malaria protection. This may be especially true for the observation that falstatin_FL_-seropositive individuals have reduced risk of progression to febrile malaria once parasitemia has been established (hazard ratio, 0.48), given the wide 95% confidence intervals (0.12–1.98). This potential association was independent of PfRH5 seropositivity and the presence of the HbAS genotype (both well-established as modifiers of malaria risk [11, 24–26]) and warrants follow-up in a larger cohort. Confirmation of this finding may support a mechanism in which anti-falstatin antibodies act in concert with antibodies against merozoite invasion ligands such as PfRH5 to reduce the risk of febrile malaria.

Our functional studies contrast with a prior study, which showed that affinity-purified anti-falstatin antibodies inhibited the formation of new rings, the earliest intraerythrocytic forms, after incubation with schizonts using falstatin-specific IgG concentrations as low as 2.5 µg/mL (9). We found no evidence that affinity-purified anti-falstatin IgG could neutralize *P. falciparum* blood stages *in vitro* in standard 40-h growth inhibitory assays using falstatin-specific IgG concentrations as high as 4 mg/mL. Using 20-h invasion assays and falstatin-specific IgG concentrations that are more faithful to the methods used by Pandey et al. (9), we did not observe any invasion inhibition by flow cytometry across multiple erythrocyte donors. However, by microscopy, high concentrations of both anti-falstatin_289–335_ and anti-falstatin_FL_ IgG inhibited new ring formation at 20 h in one of three independent experiments. Methodological differences, including variability in staining of early ring stages or erythrocyte donor variability, may account for the divergent findings. Notably, the previous study employed antibodies raised in rats, whereas our experiments utilized rabbit polyclonal IgG. Antibodies raised in different mammalian species against the same antigen may differ in epitope specificity and functional activity due to interspecies differences in immunoglobulin gene usage, repertoire diversification, and affinity maturation, which could affect inhibitory capacity (27, 28). Together, these findings suggest that falstatin-specific IgG does not inhibit merozoite invasion under the tested conditions and underscore the importance of validating functional antibody activity across assay formats and experimental systems.

This study has several limitations. The statistical power to determine whether falstatin_FL_ seropositivity reduced the time to fever after incident parasitemia was limited by the availability of plasma samples from participants who were baseline negative for *P. falciparum*. This limitation was unavoidable given that samples from these baseline-negative individuals have been extensively utilized in studies assessing predictors of *P. falciparum* infection and malaria risk. Given the minimal overlap between PfRH5-seropositive and falstatin-seropositive individuals evaluated in this study, we were not able to assess whether having the presence of both anti-PfRH5 and anti-falstatin IgG would act synergistically to reduce malaria risk or inhibit parasite growth. Lastly, our functional evaluation of anti-falstatin antibodies relied on polyclonal rabbit IgG, which may not have been optimally directed against the BC loop region that functions to inhibit cysteine proteases (15), despite one construct (falstatin_289–335_) overlapping significantly with this region.

In summary, falstatin exhibits low, natural immunogenicity in a malaria-endemic population exposed to seasonal but intense transmission. The presence of anti-falstatin IgG at baseline does not seem to predict protection against malaria overall but may be associated with reduced progression to fever once parasitemic, although this protective effect appears to be more modest than the protection conferred by sickle cell trait and naturally acquired anti-PfRH5 antibodies. Larger cohort studies would be needed to confirm whether the presence of pre-existing anti-falstatin antibodies can delay the progression of malaria symptoms after blood-stage infection.

## MATERIALS AND METHODS

### Study design and participants

The study site and study population have been previously described (10, 29). Briefly, the study was conducted in Kalifabougou, Mali, an area of intense seasonal *P. falciparum* transmission from June through December (10). In May 2011, 695 healthy children and adults (3 months–25 years of age) were enrolled into a longitudinal cohort to investigate malaria immunity. Active surveillance was performed biweekly, with additional home visits and passive case detection. Anemia was defined according to World Health Organization (WHO) criteria using hemoglobin values determined with a Hemocue 201+ analyzer (Hemocue, Angelholm, Sweden). Exclusion criteria at enrollment included a hemoglobin level <7 g/dL, axillary temperature ≥37.5°C, acute systemic illness, underlying chronic disease, use of antimalarial or immunosuppressive medications in the past 30 days, or pregnancy status. Malaria episodes were defined as parasitemia of ≥2500 parasites/μL and an axillary temperature of ≥37.5°C within 24 h, without an alternative cause. Episodes were detected prospectively through self-referral to the study clinic and weekly active clinical surveillance visits. All individuals with signs or symptoms of malaria and any level of parasitemia detected by microscopy were treated according to the Malian National Malaria Control Program guidelines. During the scheduled clinic visits, blood was collected by finger prick every 2 weeks for blood smears and dried blood spots (DBSs) on filter paper. Asymptomatic *P. falciparum* infections were detected by microscopic examination of blood smears and polymerase chain reaction (PCR) analysis of DNA extracted from dried blood spots at the end of the surveillance period. First *P. falciparum* infections were detected retrospectively by PCR of longitudinally collected DBSs, as previously described (10). First malaria episodes were determined from the clinical visit data. Hemoglobin typing for HbS was performed using a D-10 Hemoglobin Testing System (Bio-Rad).

### Detection of *P. falciparum* infections

Retrospective detection of *Plasmodium* infections was performed at the end of the surveillance period, as previously described (10, 30). In brief, for each participant, nested PCR was performed to amplify parasite DNA extracted from dried blood spots preserved on Whatman 903 Protein Saver filter paper using primers targeting the 18S ribosomal RNA gene of human *Plasmodium* species, in chronological order, until the first *P. falciparum* infection was detected.

### Genetic diversity analysis to identify the conserved regions within each target antigen

The global genetic diversity of falstatin was assessed using the MalariaGEN *P. falciparum* Community Project variant catalog, which contained 3,488 *P. falciparum* field isolates across 23 countries at the time of analysis in June 2021 (14). Briefly, variant calls for the falstatin-encoding gene were obtained from MalariaGEN, and non-synonymous polymorphisms were mapped onto the primary structure of the falstatin protein (Fig. 1A) to identify conserved regions.

### Generation of falstatin_35–68_ and falstatin_289–335_ peptides

Peptides corresponding to falstatin_35–68_ or falstatin_289–335_ were synthesized by GenScript Biotech using solid-phase peptide synthesis and purified by high-performance liquid chromatography (HPLC).

### Generation of recombinant full-length falstatin protein

Recombinant full-length falstatin without the N-terminal signal peptide (aa 22–413) was generated in *Escherichia coli* by GenScript Biotech with an N-terminal histidine-tag (His_6_), followed by a Tobacco etch virus protease (TEV) cleavage site. In parallel, constructs were synthesized without the TEV cleavage site by site-directed mutagenesis of the His_6_-TEV-falstatin_FL_ construct. His_6_-TEV-falstatin_FL_ and His_6_-falstatin_FL_ nucleotide expression constructs were derived from *Pf*3D7 and synthesized in a codon-optimized fashion with flanking sites for restriction enzymes NdeI (CATATG) and HindIII (AAGCTT). The expression constructs were cloned into pET30a expression vector and expressed in the BL21 Star (DE3) *E. coli* strain using 0.5 mM isopropyl β-D-1-thiogalactopyranoside (IPTG) induction. Recombinant protein was purified from culture supernatants using Ni-NTA affinity chromatography, followed by endotoxin removal. For His_6_-TEV-falstatin_FL_, TEV protease treatment was used to remove the His_6_ tag. Protein purity was assessed by SDS-PAGE and endotoxin testing.

### Generation of affinity-purified anti-falstatin antibodies

Immunizations, serum collection, and polyclonal antibody purification were done by Labcorp Early Development Laboratories Inc. (formerly Covance Research Products Inc.). For both constructs (falstatin_FL_ and falstatin_289–335_), two female specific pathogen-free (SPF) New Zealand White rabbits (*Oryctoloagus cuniculus*) were immunized by subcutaneous inoculation with either 500 μg falstatin_FL_ or falstatin_289–335_ together with 0.5 mL of Freund’s complete adjuvant. All animals weighed ~2.5 kg and were 5–6 months old at the time of the first immunization. Four booster doses of 250 μg protein or peptide with 0.5 mL Freund’s incomplete adjuvant were administered subcutaneously three weeks apart after the initial immunization. Test bleeds of 5 mL were performed pre-immunization and 10 days after the first booster dose. Production bleeds of 20 mL were performed 10 days after the second, third, and fourth booster doses, followed by terminal bleeds of 50 mL 13 days after the last dose. Antibody levels were tested at each bleed by ELISA. Falstatin-specific IgG antibodies were purified from serum collected at terminal bleeds and pooled from both rabbits using Protein A beads, followed by affinity purification using resin conjugated to either falstatin_289–335_ or falstatin_FL_. For 6×His falstatin_FL_, serum was additionally passed over histidine resin to remove antibodies against the His tag.

### SDS and immunoblots

Purified proteins and peptides were assessed for purity and integrity by SDS-PAGE and immunoblot. Briefly, purified protein (falstatin_FL_) and synthetic peptide (falstatin_289–335_) samples were prepared in Laemmli or Tricine sample buffer containing dithiothreitol and heated to 90°C for 5 min before loading. The falstatin_FL_ (50 ng) and falstatin_289–335_ (200 ng) were resolved using glycine-based TGX gels (Bio-Rad) and tris-tricine gels (Bio-Rad), respectively, at room temperature, following the recommended conditions. In addition, we also included lysates from uninfected erythrocytes or *P. falciparum*-infected erythrocytes on each gel. Gels were transferred onto nitrocellulose membranes (0.2 µm, Bio-Rad) using a semi-dry method (Turboblot, Bio-Rad) and confirmed by Ponceau staining. The blots were rinsed with TBS-T, blocked with 5% non-fat dry milk in TBS-T (blocking buffer) for 1 h at room temperature, and then incubated overnight at 4°C with rabbit-raised anti-falstatin_FL_ (10 µg/mL) or falstatin_289–335_ (10 µg/mL) polyclonal antibodies. Polyclonal antibodies isolated from rabbit serum collected prior to immunization were used at the same concentration as the falstatin-specific antibodies. Following primary incubation, the blots were washed and incubated with anti-rabbit H + L HRP-conjugated secondary antibodies (ImmunoReagents, Inc.) at 1:10,000 dilution for 1 h at room temperature in the dark. After washing the membrane, immunoblots were developed using enhanced chemiluminescence (Pierce). Images were acquired using the ChemiDoc MP Imaging System (Bio-Rad).

### Analysis of naturally acquired antibodies using ELISA assay

Total IgG responses to falstatin_FL_ and the conserved fragments (falstatin_35–68_ and falstatin_289–335_) were measured by ELISA using plasma from 578 cohort participants. Plates were coated overnight at 4°C with recombinant antigens (1 µg/mL; GenScript) in PBS. After washing with PBS–0.05% Tween-20 (PBST), plates were blocked with 5% nonfat dry milk in wash buffer for 2 h at room temperature. Plasma samples (1:200) were incubated for 2 h, followed by horseradish peroxidase–conjugated rabbit anti-human IgG (Dako) at 1:5,000 dilution in blocking buffer (5% nonfat dry milk in PBST) for 1 h at room temperature. Signal was developed with SureBlue substrate (SeraCare KPL) for 30 min, followed by the addition of TMB Stop Solution (SeraCare KPL). Optical density was read immediately at 450 nm with background correction at 540 nm using a SpectraMax iD3 plate reader. For subclass-specific ELISAs, HRP-conjugated mouse anti‐human IgG1 (Invitrogen), IgG2 (Invitrogen), IgG3 (Invitrogen), and IgG4 (Invitrogen) antibodies were used at dilutions of 1:1,500 against 1:100 plasma dilutions.

### Parasite culture

*Plasmodium falciparum* 3D7 (ATCC) was cultured in RPMI-1640 supplemented with Albumax II, hypoxanthine, HEPES, sodium bicarbonate, and gentamicin, using human erythrocytes at 2% or 4% hematocrit, under a gas mixture of 90% N₂, 5% CO₂, and 5% O₂ at 37°C. Synchronization was performed using 5% sorbitol treatment, with additional magnetic column-based (Miltenyi Biotec, Bergisch Gladbach, Germany) purification for tight synchrony.

### Immunofluorescence assay

Rabbit polyclonal IgG antibodies raised against falstatin_FL_ and falstatin_289–335_ were tested for recognition of native parasite proteins by IFA. *Plasmodium falciparum* schizonts purified from *in vitro* cultures using magnetic columns were smeared, air-dried, and fixed with cold methanol for 10 min. Slides were washed with PBS, blocked with PBS–1% BSA for 1 h, and incubated overnight at 4°C with rabbit anti-falstatin IgG (cross-absorbed with human erythrocytes) at 5 µg/mL. After PBS washes, Alexa Fluor 488–conjugated goat anti-rabbit IgG (Invitrogen) was applied at a 1:1,000 dilution for 1 h in the dark. Slides were incubated with Vectashield Antifade Mounting Medium with DAPI (Vector Laboratories) with coverslips for 24–48 h. Images were acquired using a Leica Thunder fluorescence microscope equipped with a Leica K5 camera and were processed for background subtraction, brightness, and contrast.

### *In vitro* growth inhibitory assay

The ability of purified rabbit polyclonal IgG to inhibit *P. falciparum* (3D7) growth was assessed by GIA as previously described (17, 31). Six serial two-fold dilutions of IgG (starting at 4 mg/mL) were incubated with late trophozoite–stage parasite cultures for ~40 h. Parasite growth was quantified by measuring *P. falciparum* lactate dehydrogenase activity. Purified IgG from PfAMA1-3D7 immunized rabbits (anti-PfAMA1 IgG, kindly provided by Kazutoyo Miura) (32) and non-immunized rabbits were included as positive and negative controls, respectively, in all assays. To directly compare with the previous study (9), affinity-purified, falstatin-specific IgG was also tested at lower doses against late trophozoite/early schizont-stage cultures for 20 h. Six serial two-fold dilutions of IgG, starting at 100 µg/mL, were used in these assays. Parasite invasion was then assessed after one replication cycle by quantifying ring-stage parasitemia using two-color flow cytometry or microscopy. For flow cytometric analysis, erythrocytes were stained with Hoechst (1:1,000) and dihydroethidium (DHE; 1:500), diluted in PBS, for 15–20 min at room temperature as previously described (33). Samples were washed two to three times with 1× PBS, resuspended in PBS, and acquired on a BD LSRFortessa flow cytometer, with 50,000 events collected per sample. Initial gating was established using uninfected erythrocytes to account for erythrocyte autofluorescence. For microscopy, thin blood smears were prepared from the same cultures, air-dried, fixed in methanol, and stained with Giemsa. Parasitemia was quantified by counting infected erythrocytes under the microscope (a minimum of 1,000 total erythrocytes counted per slide).

### Statistical analysis

Wilcoxon rank-sum test was used to compare the differences between groups for continuous outcomes. For categorical variables, group comparisons were performed using χ^2^ tests or Fisher’s exact tests as appropriate. The Kaplan–Meier curve was used to estimate the probability of remaining free of clinical malaria from the time of first *P. falciparum* blood‐stage infection as well as from the time of enrollment. For the time to clinical malaria after PCR-confirmed parasitemia, the estimated time of incident parasitemia, defined as the midpoint between the last negative *P. falciparum* PCR result and the first positive *P. falciparum* PCR result, was used as the start time. The significance of differences in time‐to‐first clinical malaria between groups was determined using the log‐rank test. Cox proportional hazards models were used to determine whether the risk of febrile malaria was associated with falstatin‐specific IgG response, using either enrollment or the time of incident *P. falciparum* blood‐stage infection as the start time. Potential confounding variables included in the model were age as a continuous variable and *Pf* PCR status at enrollment, sickle cell trait, and *Pf*RH5‐specific IgG levels as categorical variables. Statistical significance was defined as a two‐tailed *P* value of < 0.05. All analyses were performed in R version 4.4.1 (https://www.r-project.org/).

## SUPPLEMENTAL MATERIAL

Supplemental material iai.00202-26-s0001.pdf Fig. S1 to S6.

ASM does not own the copyrights to Supplemental Material that may be linked to, or accessed through, an article. The authors have granted ASM a non-exclusive, world-wide license to publish the Supplemental Material files. Please contact the corresponding author directly for reuse.
