# Supplementary material for: Sero-epidemiological and functional evaluation of IgG specific for a cysteine protease inhibitor of Plasmodium falciparum, falstatin
Source: Infect Immun. 2026 Aug 4;94(9):e00202-26. doi: 10.1128/iai.00202-26 (PMC13552103; doi:10.1128/iai.00202-26)
Supplement: Supplementary file 1 [file iai.00202-26-s0001.pdf]

SUPPLEMENTARY INFORMATION

Sero-epidemiological and functional evaluation of IgG specific for a cysteine protease inhibitor of *Plasmodium falciparum*, falstatin.

Jyoti Bhardwaj[a], Erik L. Gaskin[a], Prasida Holla[b], Oscar De Los Santos[a], Safiatou Doumbo[c], Kassoum Kayentao[c], Aissata Ongoiba[c], Boubacar Traore[c], Peter D. Crompton[d], Tuan M. Tran[a,b]

[a] Division of Infectious Diseases, Department of Medicine, Indiana University School of Medicine, Indianapolis, Indiana, USA

[b] Division of Pediatric Infectious Diseases and Global Health, Department of Pediatrics, Indiana University School of Medicine, Indianapolis, Indiana, USA

[c] Mali International Center of Excellence in Research, University of Sciences, Technique and Technology of Bamako, Bamako, Mali.

[d] Malaria Infection Biology and Immunity Section, Laboratory of Immunogenetics, National Institute of Allergy and Infectious Diseases, National Institutes of Health, Rockville, Maryland, USA.

# Address correspondence to Tuan M. Tran, tuantran@iu.edu.

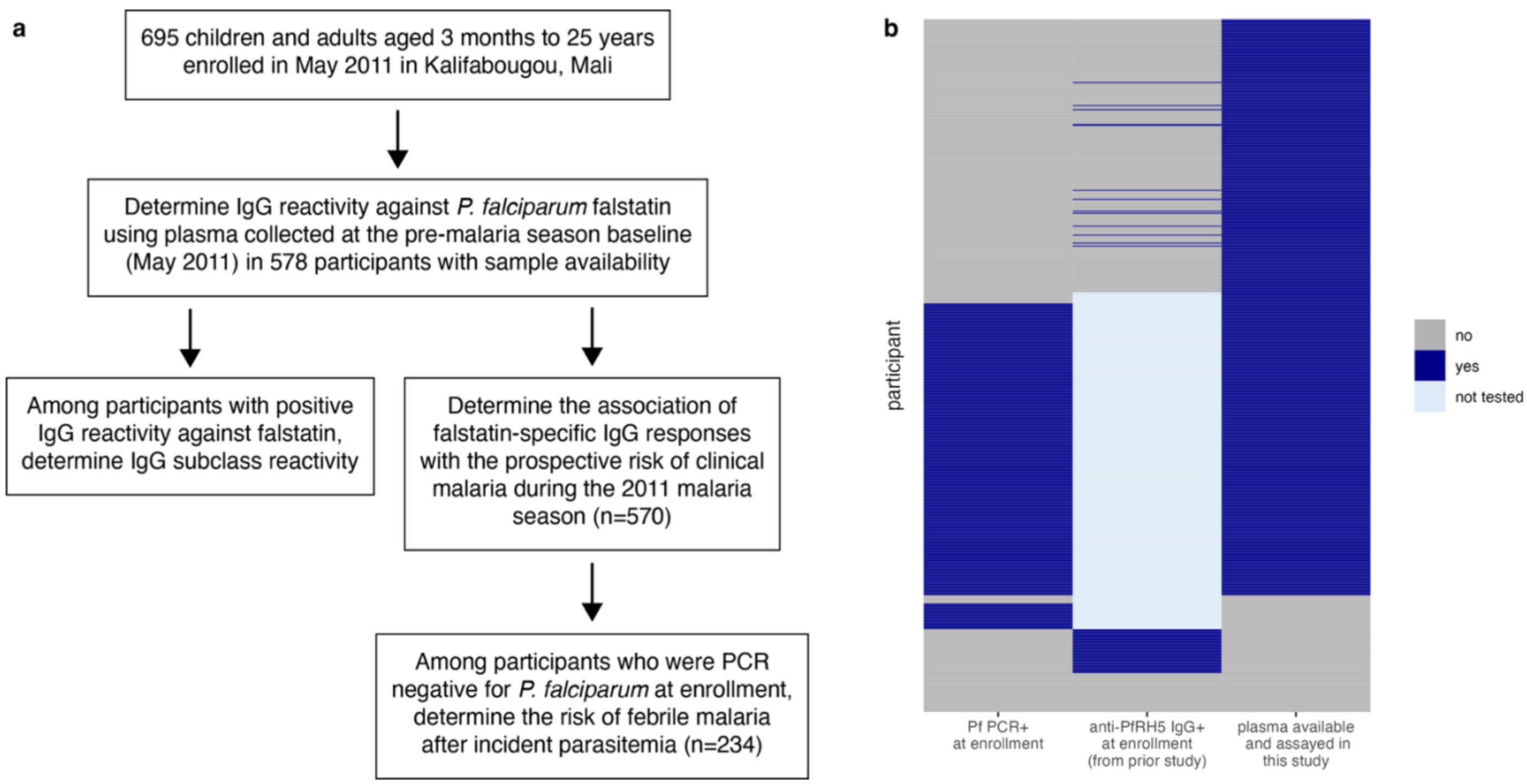

Figure S1. Overall design for seroepidemiological studies. (A) Flow-diagram of study design and analyses. **(B)** Availability of plasma samples for current study (n=578) in relation to the total participants from the original Kalifabougou cohort (n=695) by *Pf* PCR status and PfRH5-specific IgG seropositivity status at enrollment.

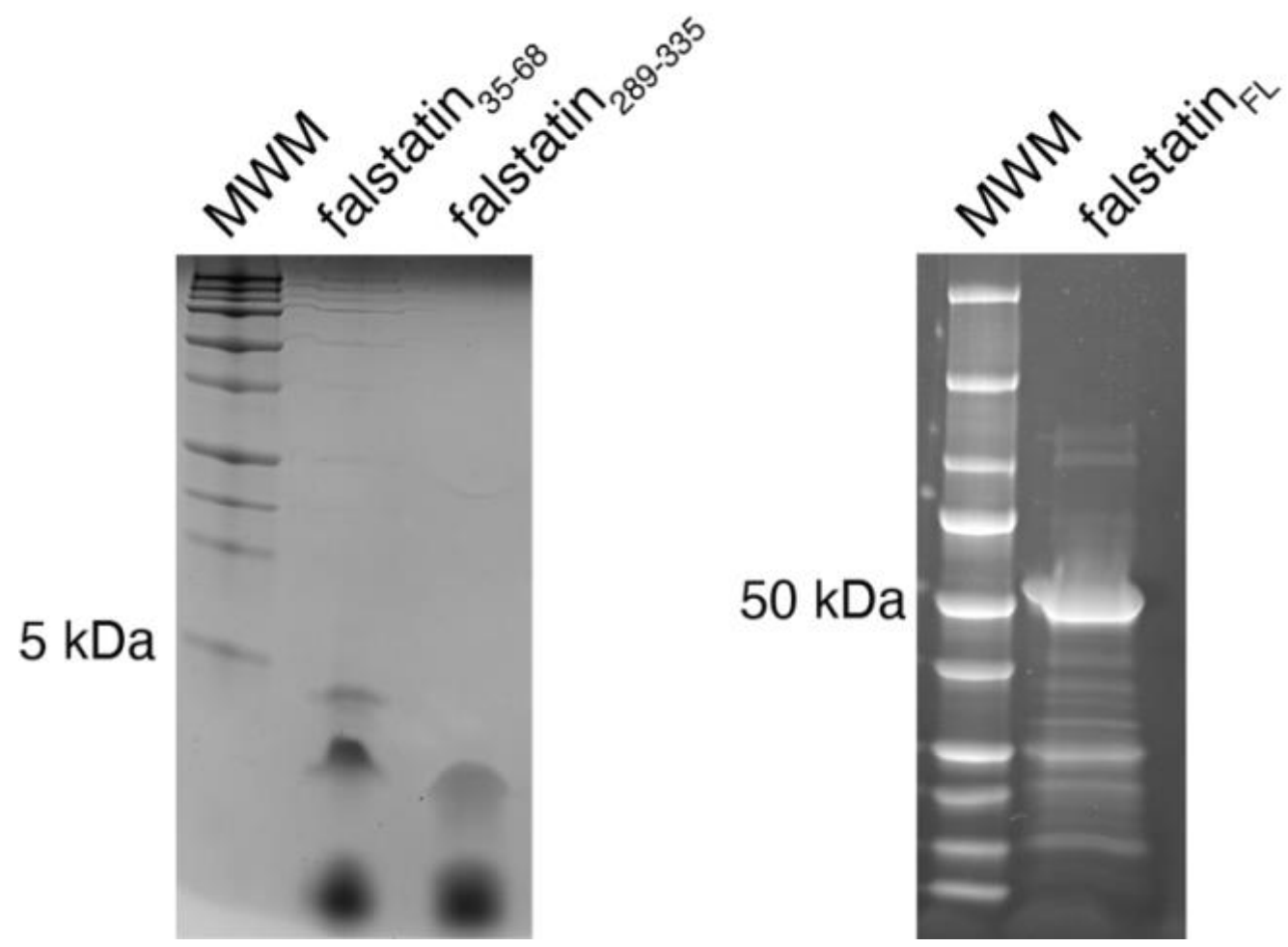

Figure S2. Falstatin constructs resolved by gel electrophoresis. Approximately 10 µg of polypeptide or protein were resolved by sodium dodecyl sulfate–polyacrylamide gel electrophoresis (SDS-PAGE) under reducing and denaturing conditions. Polypeptides were resolved using Tris-tricine-SDS-PAGE whereas recombinant protein (falstatin$_{FL}$) was resolved using Tris-glycine-SDS-PAGE.

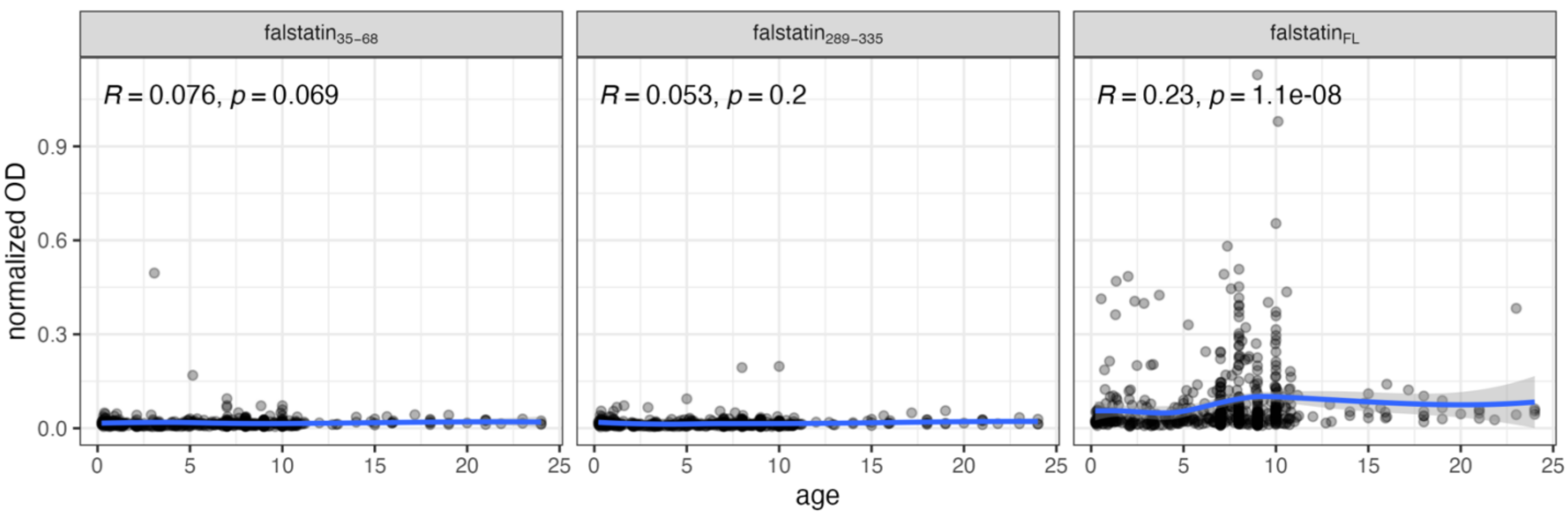

antigen	n	Spearman rho	lower 95% CI	upper 95% CI	p-value	adjusted p-value
falstatin[35-68]	578	0.07568	-0.005909	0.1563	6.903e-02	2.071e-01
falstatin[289-335]	578	0.05337	-0.028300	0.1343	2.001e-01	6.002e-01
falstatin[FL]	578	0.23460	0.156000	0.3102	1.145e-08	3.435e-08

Figure S3. Relationship between falstatin-specific IgG responses and age. Scatter plots showing IgG reactivity against the indicated falstatin antigens versus age (years) at enrollment. Locally weighted scatterplot smoothing (LOWESS) curves (blue lines) with 95% confidence bands are shown to capture nonlinear trends. R = Spearman rho. OD = optical density. CI = confidence interval. Related to Figure 1B.

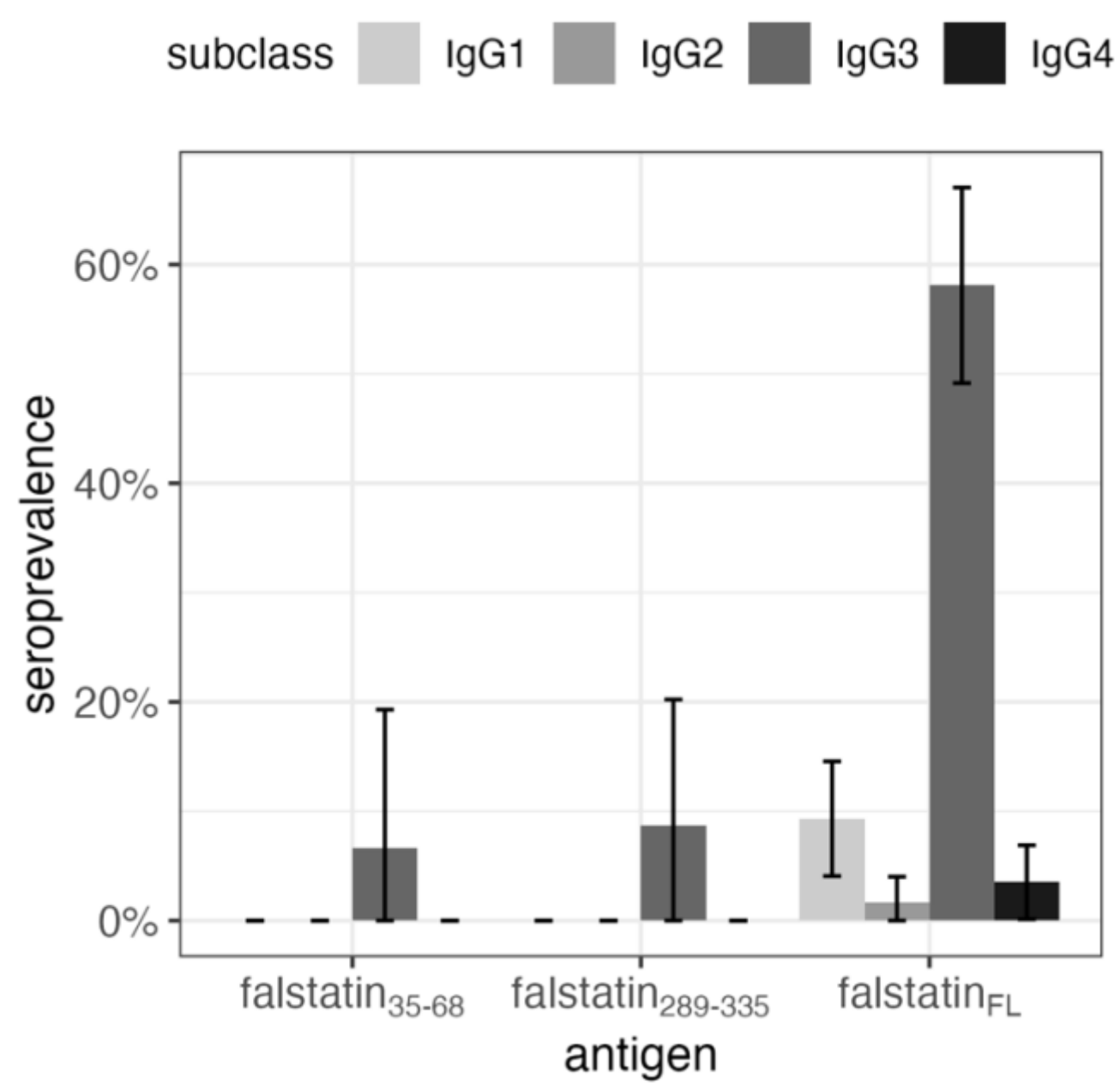

Figure S4. IgG subclass reactivity. Percentage of participants (seroprevalence) who demonstrated IgG1, IgG2, IgG3, or IgG4 subclass reactivity to each falstatin region among those who demonstrated total IgG seropositivity for the same region. A seropositive response was considered to be ≥3 standard deviations above the mean reactivity of 14 malaria-naive US donors. Error bars are 95% confidence intervals using a normal distribution approximation.

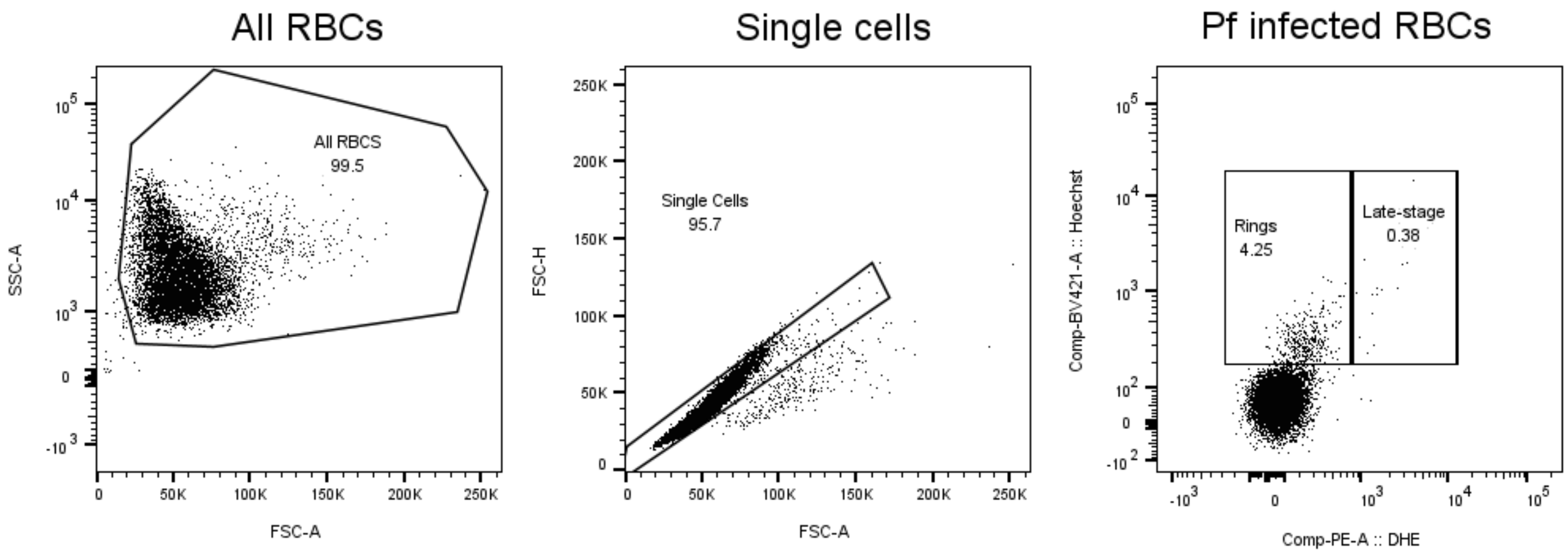

Figure S5. Gating strategy for 20 hour invasion assay by flow cytometry.

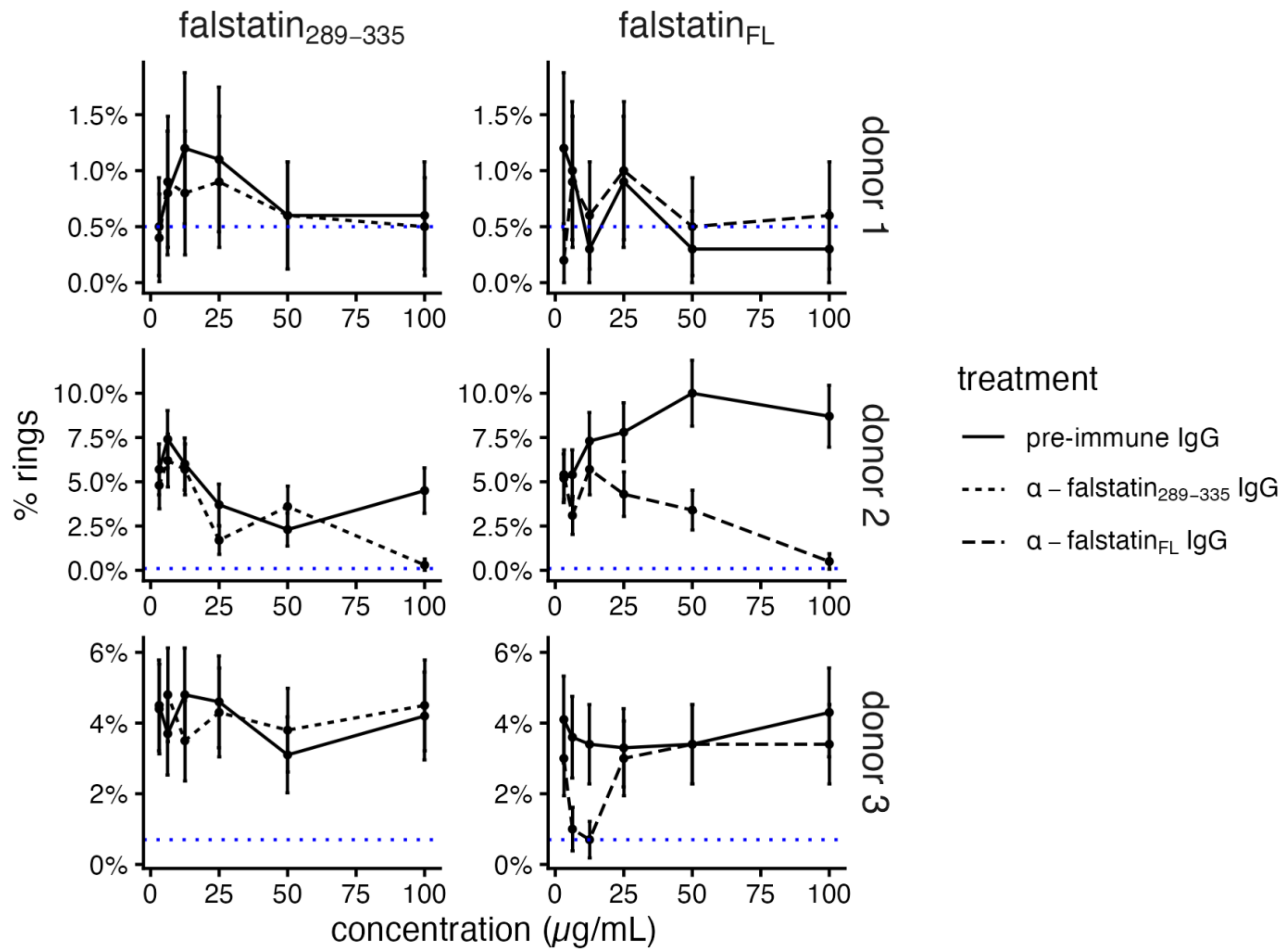

Figure S6. Results of 20-hour ring invasion assay by microscopy. Late trophozoite/early schizont-stage *P. falciparum* (3D7) cultures were incubated with serial two-fold dilutions of IgG for 20 h. Parasite invasion was assessed by quantifying ring-stage parasitemia by microscopy. Shown are 95% confidence intervals using the normal approximation. Blue horizontal dotted line represents the % rings for α-PfAMA1 IgG (total IgG purified from PfAMA-immunized rabbits) at 1 mg/mL. Data are from independent experiments with each using erythrocytes from a different donor.
